# Vapor–Liquid Equilibria Data for 2-Piperidineethanol and 1-(2-Hydroxyethyl)pyrrolidine in Aqueous Solutions and a UNIQUAC Model Representation

**DOI:** 10.1021/acs.jced.1c00726

**Published:** 2022-01-04

**Authors:** Ardi Hartono, Christina Nøkleby, Inna Kim, Hanna K. Knuutila

**Affiliations:** † Department of Chemical Engineering, Norwegian University of Science and Technology, N-7491 Trondheim, Norway; ‡ SINTEF Industry, P.B. 4760, 7465 Trondheim, Norway

## Abstract

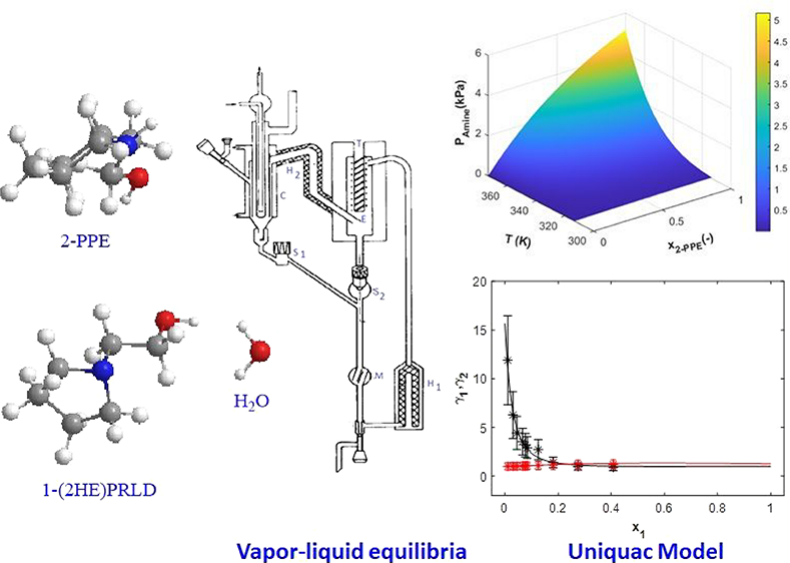

This work reports equilibrium data for two amines, 2-piperidineethanol (2-PPE) and 1-(2-hydroxyethyl)pyrrolidine (1-(2HE)PRLD), and their aqueous solutions. The pressure, temperature, and composition data are used to calculate experimental activities. Data cover temperatures from 363 to 426 K for the pure amines and from 323 to 373 K for the aqueous solutions. A UNIQUAC model was used to represent the binary vapor–liquid equilibria (VLE), whereas the Antoine equation was used for pure components. In an aqueous solution, the vapor pressure of 1-(2-hydroxyethyl)pyrrolidine (1-(2HE)PRLD) over the measured composition and temperature ranges is higher than that of 2-piperidineethanol (2-PPE). The developed UNIQUAC models represent the data well. For 2-piperidineethanol (2-PPE), the model gave 1.9% deviations for total pressure, 12.4% for vapor-phase composition, 12.7% for the calculated activity coefficients, and 16.2% for the excess heat capacity. In the case of 1-(2-hydroxyethyl)pyrrolidine (1-(2HE)PRLD), the model was slightly more accurate, representing the data with 1.7% deviation for total pressure, 5.9% for vapor-phase composition, and 5.2% for the calculated activity coefficient.

## 1 Introduction

Human activities mainly cause increasing carbon dioxide (CO_2_) concentrations in our atmosphere. The CO_2_ level has increased up to 430 ppm, a close to 50% increase compared to the last century. The increasing CO_2_ level links to global warming, and there is a need to cut the CO_2_ emissions.^1^ One of the most efficient technologies to reduce carbon dioxide emissions is using chemical-absorption-based technologies to capture CO_2_ and send the produced pure CO_2_ for underground storage. CO_2_ capture with chemical absorption is a mature technology, with drawbacks like high energy consumption and solvent volatility, increasing operating costs, and solvent emissions. Installing water-wash or acid-wash sections on top of the absorber reduces solvent emissions. However, the design of these columns requires knowledge about the equilibrium for amine and the water system.

Aqueous alkanolamine solutions are widely used as solvents for CO_2_ capture from various gas streams, and understanding the amine volatility allows the design of the CO_2_ capture process with minimum amine emissions. We have previously reported screening results of strong bicarbonate-forming solvents for CO_2_ capture,^2^ characterization of selected solvent systems,^3^ and evaluation of process performance using 1-(2-hydroxyethyl)pyrrolidine (1-(2HE)PRLD) and 2-piperidineethanol (2-PPE)^4^ for postcombustion CO_2_ capture. There is no volatility data for 2-PPE, but some data for 1-(2HE)PRLD are recently reported for temperatures between 353 and 373 K.^5^

Vapor–liquid equilibrium measurement can be performed using either static or dynamic apparatuses. In the static device, vapor pressure is measured in a closed vessel (with constant volume) at a constant temperature.^6^ The liquid composition can be determined from the initial liquid fed into the cell vessel or by analyzing liquid samples. This type of experiment produces typically *PTx* data, and vapor-phase composition is estimated from a thermodynamic model.^7−9^ An ebulliometer can provide dynamic measurements of *PTx* or *PTxy* data for pure, binary, and ternary systems. The *PTxy* data may then be used to calculate the experimental activity coefficients of the components.^10^

In the current work, we report on ebulliometer measurements of two amines, 2-piperidineethanol (2-PPE) and 1-(2-hydroxyethyl)pyrrolidine (1-(2HE)PRLD), and their aqueous mixtures. VLE data were measured up to mole fractions of 0.40 between 323 and 373 K for aqueous solutions. For pure chemicals, the VLE was measured between 363 and 426 K. Finally, the experimental data were modeled using the UNIQUAC framework.^11^

## 2 Experimental Section

### 2.1 Chemicals and Procedures

Two commercially available chemicals from TCI Chemicals, 2-piperidineethanol (2-PPE) and 1-(2-hydroxyethyl)pyrrolidine (1-(2HE)PRLD), were used as received without any further purification, as seen in Table 1.

**Table 1:**
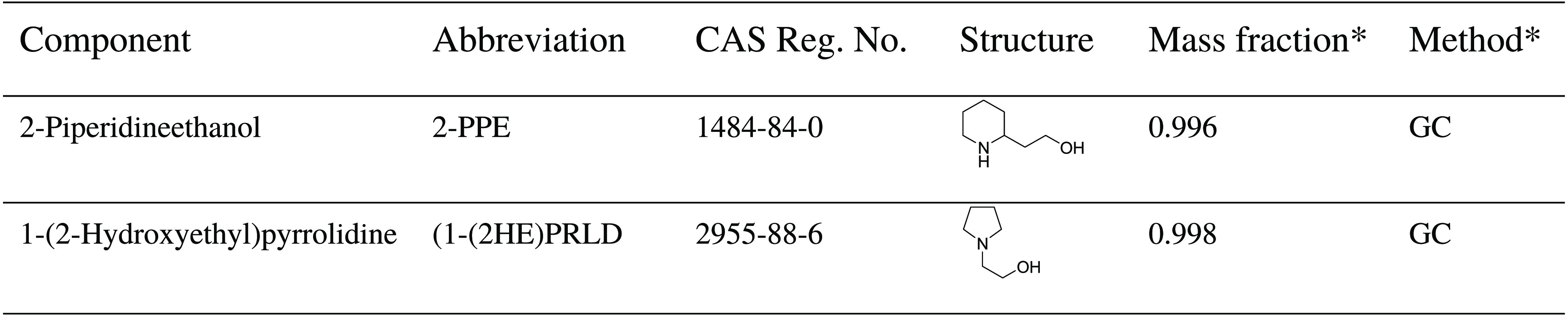
Chemical Used

* The Certificate of Analysis (CoA).

2-PPE is in the solid phase at ambient conditions, while 1-(2HE)PRLD is a clear liquid. As the melting point of 2-PPE is 36 °C according to the supplier,^12^ the chemical was kept in a tightly closed bottle in a heating cabinet at ∼45 °C. During pure component measurements, the 2-PPE was completey melted and transferred directly into the ebulliometer. No special treatment for pure 1–2(HE)PRLD was needed since it is liquid at room temperature. Aqueous amine solutions of different concentrations were prepared gravimetrically using distilled deionized water. Solutions up to 85 mass % of 2-PPE (∼0.44 mole fraction) and up to 80 mass % of 1-(2HE)PRLD (∼0.40 mole fraction) in deionized water were prepared.

The VLE of the two amines were measured with a modified Swietoslawski ebulliometer,^13^ earlier shown by refs (14−18). The experiments were started by feeding about 0.9 dm^3^ of the solution of interest into the ebulliometer through a sampling port. External heaters equipped with temperature sensors were used to increase the liquid temperature. The system was considered to be in equilibrium when the boiling was even; i.e., the pressure and temperature were stable, and condensate droplets were continuously produced. Experimental temperature and pressure were logged using LabVIEW software via a Chub-E4 thermometer readout (Hart Scientific, Fluke) and a pressure controller of type DPI520 (Druck, Germany). The temperature and pressure of the equilibrated system were measured for the pure compounds. In the case of binary mixtures, in addition to the pressure and temperature data, liquid- and gas-phase sampling was done. Approximately up to 2 cm^3^ of both liquid and vapor phases was taken out as samples.

### 2.2 Sample Analysis

The collected liquid and vapor samples were analyzed by an acid–base titration technique (Mettler Toledo G20) with sulfuric acid as titrant. Two stocks of H_2_SO_4_ solutions were prepared, i.e., 0.1 mol/dm^3^ (∼0.976 mass %) for high concentration samples and 0.01 mol/dm^3^ (∼0.098 mass %) for low concentration samples. The lowest concentrations that can be analyzed with the 0.01 mol/dm^3^ H_2_SO_4_ solution were about 0.0002 in mole fraction. Duplicate samples were titrated and with typical deviations less than 3%. If the analyzed concentration was lower than 0.0002 in mole fraction, the sample was then analyzed with a Cation Chromatography (IC) (Thermo Scientific Dionex ICS-500) using an existing method.^19^

### 2.3 Sources of Uncertainties

Temperature, pressure, and analytical methods for composition determination are the three main sources of uncertainty. The liquid-phase solvent concentration, *x*, and vapor-phase concentration, *y*, contribute to the experimental uncertainty more than the measured temperature, *T*, and pressure, *P*. Uncertainties of the measured pressure were estimated similarly to the work of Schiering and Schnelle-Werner,^20^ and for the measured concentration by IC, the work of Leiva et al.^21^ was used. The values are given in the tables together with experimental results. The calculated standard uncertainties of the liquid-/vapor-phase analysis suggest that *u*(*x*) = *u*(*y*) = 0.0002 in mole fraction units and *u*(*x*) = *u*(*y*) = 0.00003 with the ion chromatography method, respectively. In the case of experiments with pure amines, the presence of inert substances in the pure amines can significantly affect the results of the boiling point measurements if they have higher volatility than the amine (e.g., water).

### 2.4 Parameter Fitting

In the case of a pure chemical, the measured *PT* data were fitted to an Antoine correlation^22^
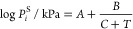
1where *P*_*i*_^S^ = saturated vapor pressure of the considered substance in kPa; *T* = temperature in K; and *A*, *B*, *C* = constants (fitted parameters).

The binary VLE data were fitted to a UNIQUAC (Universal Quasi Chemical) model^11^ based on the excess Gibbs energy described as
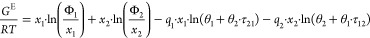
2where Φ_*i*_, θ_*i*_ and τ_*ij*_ are calculated from
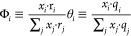

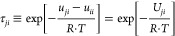
In eq 2, *x*_*i*_ = liquid mole fraction; *r*_*i*_ and *q*_*i*_ are properties related to the pure-component volume and area UNIQUAC parameters; and τ_*ij*_ = temperature-dependent binary interaction parameters.

The binary interaction parameters (*U*_*ji*_), with two fitted parameters *a*_*ij*_ and *b*_*ij*_ as proposed by Thomsen et al.^23^ were used:
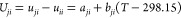
The UNIQUAC activity coefficients in the binary system were derived from the excess Gibb’s energy and expressed as^11^
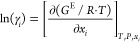

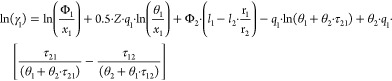
where *l*_1_ and *l*_2_ are expressed as
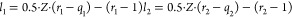
From the *PTxy* VLE data, experimental activity coefficients can be estimated according to
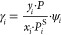
3where *y*_*i*_ = vapor mole fraction; *P*_*i*_^S^ = saturation pressure; *P* = total pressure; and ψ_*i*_ = the poynting factor. When *PTxy* VLE data are measured at low to moderate pressures, the Poynting factor has minor importance. In this work VLE measurements were performed at or below atmospheric conditions. Thus, the Poynting factor was set to unity.

In the binary system, eight parameters are present. Four of them describing the pure-component volume (*r*_*i*_) and area (*q*_*i*_) were estimated from the van der Waals volume and radii.^24^ For water, the values were taken from the literature.^11^ The values are shown in Table 2.

**Table 2:** Size (*r*) and Surface Molecule (*q*) Parameters for the UNIQUAC Model

substance	*r*_*i*_	*q*_*i*_	remarks
2-piperidineethanol	5.56	4.45	estimated based on the Bondi’s method^24^
1-(2-hydroxyethyl)pyrrolidine	4.86	3.92	estimated based on the Bondi’s method^24^
H_2_O	0.92	1.40	(11)

The other four unknown binary interaction parameters in the UNIQUAC model together with three unknown parameters in the Antoine correlation were fitted simultaneously using the new measured and available literature data. The objective function used in the fitting is given in eq 4:
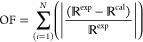
4

In the equation,  represents the responses of the experimental or modeled result. The responses were boiling points/saturation pressures, *P*_*i*_^S^, of pure compounds, total pressures, *P*_*i*_, of binary systems, vapor-phase composition, *y*_*i*_, excess enthalpy, *H*_*i*_^E^, and excess heat capacity, *Cp*_*i*_^E^, data. A Global-Search algorithm^25^ with a scatter-search mechanism for generating starting points was used.

An absolute average relative deviation (AARD) and a mean absolute deviation (MAD) were calculated with the equation
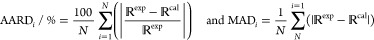
5where *N* = number of the data.

As mentioned above, the binary interaction parameters in the UNIQUAC model and parameters for the Antoine equation were fitted simultaneously using the PT data for pure amines and binary VLE data for aqueous solutions. In the case of 2-PPE, excess heat capacities for different concentrations (0.2, 0.4, 0.6, and 0.8 in mole fraction units), and temperatures (303–353 K),^26^ the data measured in this work and the normal boiling point of 2-PPE (507.2K) reported by the supplier were used in the fitting. Data from two literature sources and experimental data from this work were used in fitting the 1-(2HE)PRLD/H_2_O system.

## 3 Results and Discussion

### 3.1 Pure Amines

**Figure 1:**
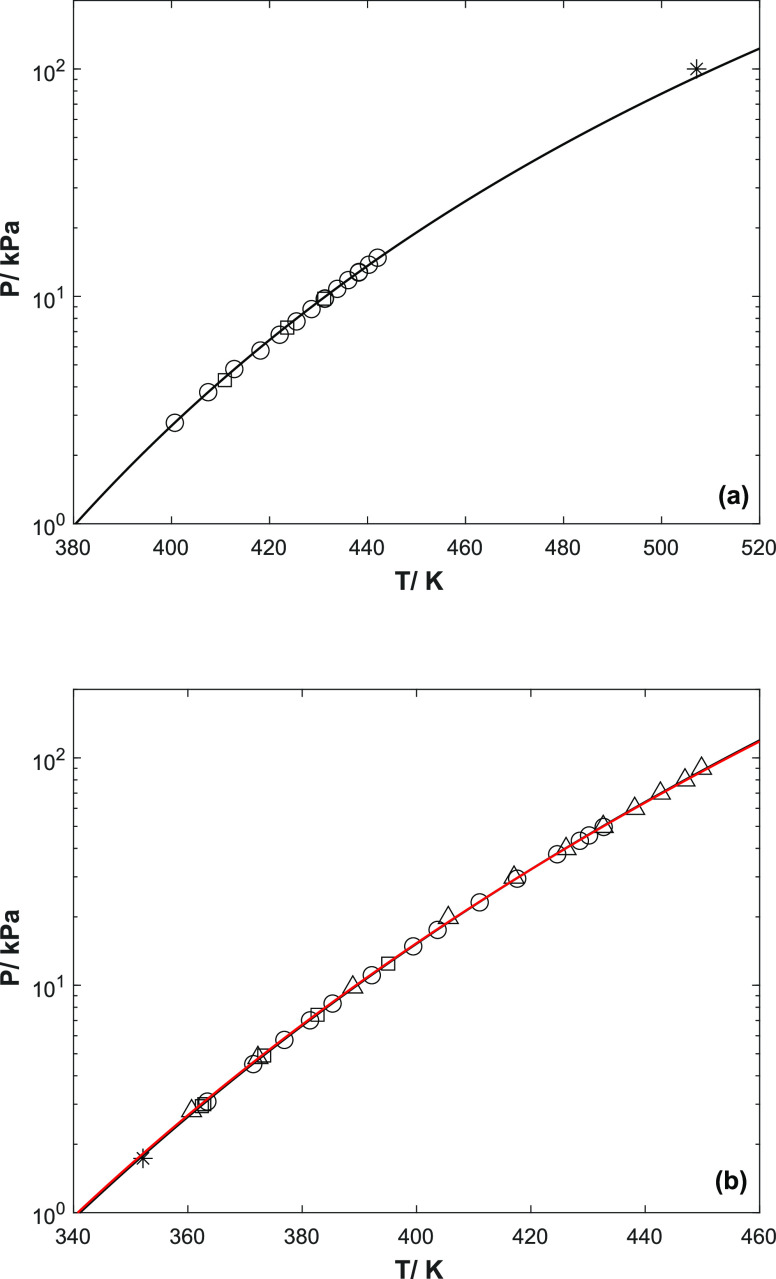
Vapor pressure of pure amines (a) 2-PPE and (b) 1-(2HE)PRLD as a function of temperature (○, exp.#1; □, exp#2; Δ, ref (5); ∗, a normal boiling point of 2-PPE;^12^**∗**, boiling point of 1-(2HE)PRLD at 1.73 kPa;^27^ black solid line, a combined fit; red solid line, ref (5)).

Table 3 reports the experimental results for boiling points of the pure substances, and Figure 1 shows the experimental results together with the Antoine equation. The parameters for the Antoine equation and the AARD values are given in Table 4.

**Table 3:** Measured Boiling Points of Pure Chemicals at Temperature *T* and Pressure *P* for 2-Piperidineethanol and 1-(2-Hydroxyethyl)pyrrolidine^a^

2-PPE			1-(2HE)PRLD		
*T*/K	*P*/kPa	*u*(*P*)	*T*/K	*P*/kPa	*u*(*P*)
Exp. 1					
400.7	2.78	0.01	362.5	2.91	0.01
407.5	3.79	0.01	363.5	3.09	0.01
412.9	4.79	0.01	371.5	4.49	0.01
418.2	5.78	0.01	376.9	5.75	0.01
422.1	6.78	0.01	381.4	7.01	0.01
425.5	7.76	0.01	385.4	8.31	0.01
428.6	8.79	0.01	392.2	11.07	0.01
431.3	9.76	0.01	399.5	14.83	0.01
431.4	9.79	0.01	403.7	17.52	0.01
433.9	10.79	0.01	411.1	23.15	0.04
436.1	11.79	0.01	417.6	29.43	0.05
438.3	12.73	0.01	424.7	37.68	0.06
438.3	12.80	0.01	428.6	43.18	0.07
440.3	13.78	0.01	430.2	45.60	0.07
442.1	14.79	0.01	432.9	49.69	0.08
Exp. 2					
410.9	4.28	0.01	362.9	2.99	0.01
423.7	7.29	0.01	373.4	4.91	0.01
431.2	9.77	0.01	382.7	7.42	0.01
-	-	-	395.1	12.45	0.01

a Standard uncertainty of *u*(*T*) = 0.1 K.

**Table 4:** Fitted Antoine Parameters for 2-Piperidineethanol (2-PPE) and 1-(2-Hydroxyethyl)pyrrolidine (1-(2HE)PRLD) together with Absolute Average Relative Deviations (AARD) and Mean Absolute Deviation (MAD)^a^

amine	A	B	C	AARD/%	MAD/kPa
2-PPE	5.6064	–1316.5607	–145.6090	1.7	0.5
1-(2HE)PRLD	8.0356	–2741.01	–0.00018	1.2	0.3

a A, B, and C are constants.

The AARD values, given in Table 4, show that the Antoine equation can be used to represent the vapor pressure of 2-PPE and 1-(2HE)PRLD with high accuracy. The Antoine equation covers a temperature range of 350–450 K for 1-(2HE)PRLD and 400–510 K for 2-PPE. Furthermore, Figure 1 shows that 1-(2HE)PRLD is more volatile than 2-PPE. The reproducibility of the measured data in this work is good, and the data agree well with Bernhardsen et al. (2019).^5^ The trend of these two sets of data also agrees with a point reported by Astle and Weast (1985),^27^ as shown in Figure 1b.

### 3.2 Binary VLE of a 2-Piperidineethanol/Water System

Table 5 lists the measured binary VLE of 2-PPE(1)/H_2_O(2) data. The experiments were done at four temperatures and amine concentrations up to ∼0.45 in mole fraction units. The results show that the amine concentration in the vapor phase is very low (∼0.003 in mole fraction units at given maximum concentration in the liquid phase (0.4129) and temperature (373 K)) due to the low volatility of 2-PPE.

**Table 5:** Experimental (Vapor + Liquid) Equilibrium Data at Temperature *T*, Pressure *P*, Liquid-Phase Mole Fraction *x*, and Vapor-Phase Mole Fraction *y*, for 2-Piperidineethanol (1) + H_2_O (2)^a^

*T*/K	*P*/kPa	*u*(*P*)	*x*_1_	*y*_1_	γ_1_^exp.^ (eq 3)	*U*(γ_1_^exp.^)
333.2	19.99	0.01	0.0007	0.00002^b^	14.81	16
333.2	19.79	0.01	0.0110	0.00022^b^	10.25	2
333.2	19.40	0.01	0.0658	0.0005	4.13	0.7
333.2	19.31	0.01	0.0804	0.0005	3.24	0.5
333.2	19.27	0.01	0.0927	0.0005	2.91	0.5
333.4	19.05	0.01	0.1310	0.0008	2.83	0.4
333.2	18.50	0.01	0.1748	0.0006	1.76	0.3
333.2	17.39	0.01	0.2755	0.0006	0.98	0.2
333.2	13.78	0.01	0.4535	0.0013	0.97	0.2
343.2	31.18	0.05	0.0007	0.00003^b^	15.74	15
343.2	30.87	0.05	0.0104	0.00037^b^	12.61	7
343.2	30.47	0.05	0.0452	0.0006	4.55	3
343.2	30.28	0.05	0.0664	0.0007	3.95	2
343.2	30.20	0.05	0.0787	0.00090^b^	3.60	2
343.2	28.99	0.05	0.1848	0.00096^b^	1.72	1
343.2	27.10	0.04	0.2886	0.00075^b^	0.81	0.5
353.2	47.01	0.07	0.0102	0.00047^b^	24.90	5
353.2	46.48	0.07	0.0295	0.0007	13.14	2
353.2	46.26	0.07	0.0446	0.0008	9.26	2
353.2	46.09	0.07	0.0662	0.0009	7.41	1
353.2	45.87	0.07	0.0764	0.0010	6.66	1
353.2	45.82	0.07	0.0835	0.0010	6.09	1
353.2	45.08	0.07	0.1250	0.0014	5.69	1
353.2	44.00	0.07	0.1810	0.0011	2.95	0.5
353.2	40.97	0.06	0.2747	0.0012	2.06	0.4
353.2	33.78	0.05	0.4075	0.0020	1.87	0.3
373.2	100.09	0.16	0.0103	0.00117^b^	17.21	4
373.2	99.56	0.16	0.0302	0.0013	6.38	1
373.2	98.86	0.16	0.0449	0.0014	4.46	1
373.2	98.47	0.16	0.0651	0.0016	3.64	1
373.2	98.21	0.16	0.0777	0.0015	2.91	1
373.2	97.98	0.15	0.0853	0.0015	2.57	1
373.2	96.56	0.15	0.1320	0.0017	2.03	0.5
373.2	96.56	0.15	0.1322	0.0018	1.84	0.4
373.2	94.28	0.15	0.1864	0.0019	1.43	0.3
373.2	87.79	0.14	0.2808	0.0021	0.97	0.2
373.2	71.89	0.11	0.4129	0.0032	0.84	0.2

a Standard uncertainties *u* are *u*(*T*) = 0.1 K, *u*(*x*_1_) = 0.0002, *u*(*y*_1_) = 0.0002, and *u*(*y*_1_^*^) = 0.00003.

b IC analysis.

As mentioned earlier, in addition to the pure and binary VLE data, the excess heat capacity data^26^ were also used for fitting the UNIQUAC parameters. The calculated optimum parameters are given in Table 6 together with the AARD’s values. The parity plots for all the responses are given in the Supporting Information (Figure S1).

**Table 6:** UNIQUAC Binary Interaction Parameters for 2-Piperidineethanol (1) + H_2_O (2) and 1-(2-Hydroxyethyl)pyrrolidine (1) + H_2_O (2)

parameters	2-PPE (1) + H_2_O (2)	1-(2HE)PRLD (1) + H_2_O (2)
*a*_1,2_ = *u*_1,2_	288.0637	212.4938
*b*_1,2_ = *u*_1,2_^*T*^	–1.6949	0.3167
*a*_2,1_ = *u*_2,1_	–176.3375	–212.9474
*b*_2,1_ = *u*_2,1_^*T*^	0.6105	0.6839
objective function OF_min_	0.08	0.02

AARD_*i*_, % (MAD_*i*_)		
pressure *P*	1.9 (0.78)	1.7 (0.99)
vapor-phase *y*	12.4 (0.0001)	5.9 (0.001)
excess heat capacity *C_p_*^E^	16.2 (0.99)	not available

**Figure 2:**
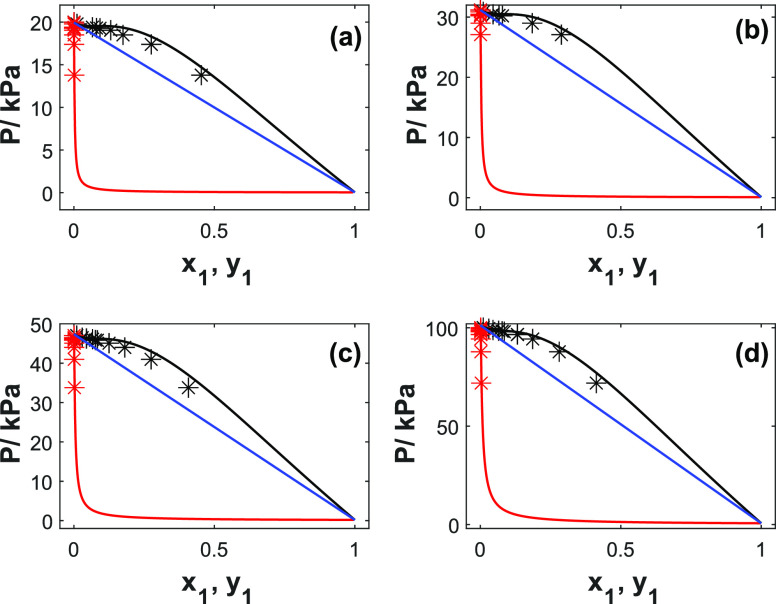
*PTxy* profiles for 2-PPE (1)/H_2_O (2) at different temperatures: (a) 333 K, (b) 343 K, (c) 353 K, and (d) 373 K. Points, data; solid lines, UNIQUAC; black lines, bubble points; red lines, dew points lines; blue lines, Raoult’s law.

Figure 2 represents the fitted UNIQUAC model for the 2-PPE (1)/H_2_O (2) system and Raoult’s law showing the ideal behavior. It may be seen that the UNIQUAC model represents the *PTxy* well (AARD within 2%). However, at higher concentrations (*x*_1_ > 0.1 in mole fraction units), a slight discrepancy is observed for the bubble point curves. The dew point curves show very low pressures already at low concentrations, indicating that the system has very low volatility. The water vapor pressure contributes predominantly to the total pressure. The measured system behaves as a nonideal solution with a positive deviation from Raoult’s law in the whole range of amine concentrations. The calculated value of the interaction energy (*U*_*ji*_) between the like molecules and unlike molecules given in Table 6 shows that the interaction between amine–amine is stronger than that of amine–H_2_O and H_2_O–H_2_O. The UNIQUAC model represents well the vapor pressure data with an AARD value of 12.4%. The figure showing the vapor-phase composition can be found in the Supporting Information (Figure S2).

**Figure 3:**
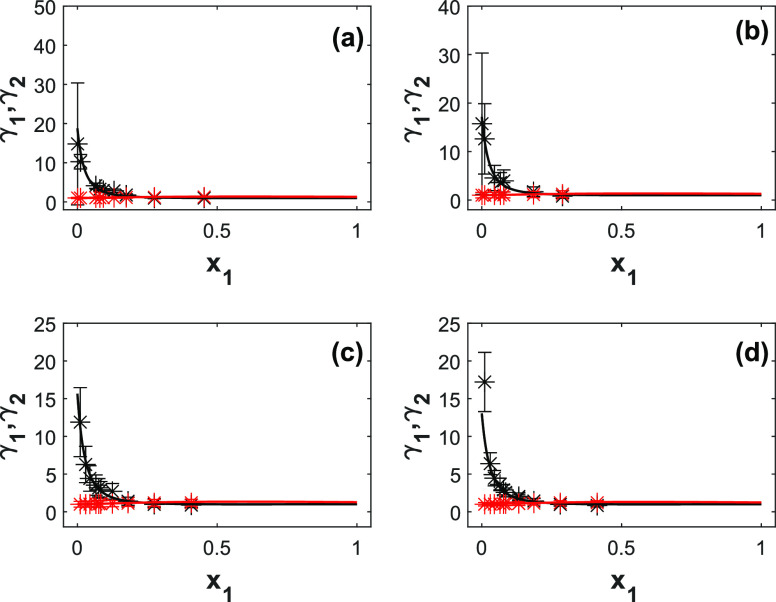
Model representation of the activity coefficients for 2-PPE (1)/H_2_O (2) at different temperatures. (a) 333 K; (b) 343 K; (c) 353 K; (d) 373 K. Point, experimental activity coefficient; solid lines, UNIQUAC; black lines, amine; red lines, water.

Figure 3 shows the experimental and modeled activity coefficients for 2-PPE (1) and H_2_O (2) as a function of liquid composition and temperature. The UNIQUAC model predicts the activity coefficients with an AARD of 12.7%. The AARD of the activity coefficient of amine, γ_1_, is slightly higher than the AARD value of amine vapor-phase composition, *y*_1_, due to the uncertainties in the pressure measurements. The activity coefficient of the amine is weakly temperature-dependent (see the *b*_*i,j*_ values in Table 6). The activity coefficients at infinite dilution of 2-PPE decrease slightly with temperature, i.e., from 18 at 333 K to 12 at 373 K. The activity coefficient of water remains unchanged. As seen in Figure 3, at lower concetrations, the estimated uncertainty is higher due to lower accuracy of the analytical method used.

**Figure 4:**
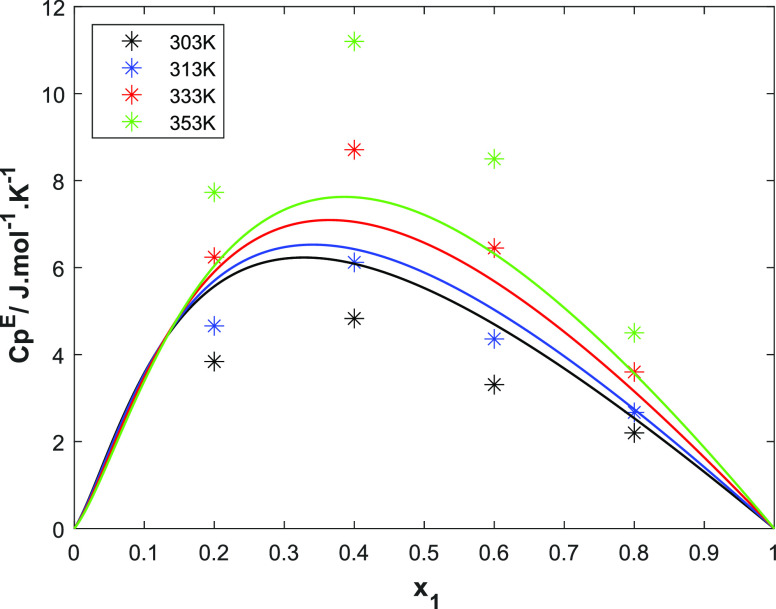
Representation of the UNIQUAC model for the excess heat capacity for 2-PPE (1)/H_2_O (2) at different temperatures (points, ref (26); solid lines, UNIQUAC).

Figure 4 compares the UNIQUAC model and the excess heat capacity data.^26^ The excess heat capacity increases with amine concentration and reaches a maximum value at 0.4 in mole fraction of amine. The excess heat capacity also increases with temperature. The excess heat capacity correlation is a second derivative of the excess Gibb’s energy with respect to temperature (shown in the Supporting Information). Overall, the model predicts the 313 K data well even though it overpredicts at low temperatures and underpredicts at higher temperatures. The modeled maximum values of the excess heat capacity are slightly shifted to lower concentrations compared to the data. Several attempts were made to improve the fit with different initial parameters and boundary values.

### 3.3 Binary VLE of the 1-(2-Hydroxyethyl)pyrrolidine (1)/H_2_O (2) System

The measured binary VLE data for 1-(2HE)PRLD (1)/H_2_O (2) are presented in Table 7 together with the experimental activity coeefficients with their uncertainty values. At lower concetrations, the estimated uncertainty is higher due to lower accuracy on the analytical method used. Four temperatures and up to ∼0.40 in mole fraction of amine in solution were measured. Only a few points at 323 K were collected due to very low pressure. The new data cover a larger range of concentrations, especially at lower concentrations than those reported previously.^5^ The low concentration data are crucial in determining the activity coefficient at infinite dilution. The partial pressure of 1-(2HE)PRLD over its aqueous solution is higher than that of 2-PPE at the same conditions. At a liquid-phase mole fraction of ∼0.4 and at 373 K, the 1-(2HE)PRLD concentration in the vapor phase is about ten times higher than that of the 2-PPE. Table 5 lists the optimum regressed parameters, and the parity plots are available in the Supporting Information (Figure S3).

**Table 7:** Experimental (Vapor + Liquid) Equilibrium Data at Temperature *T*, Pressure *P*, Liquid-Phase Mole Fraction *x*, and Vapor-Phase Mole Fraction *y*, for 1-(2-Hydroxyethyl)pyrrolidine (1) + H_2_O (2)^a^^,^^b^

*T*/K	*P*/kPa	*u*(*P*)	*x*_1_	*y*_1_	γ_1_^exp.^ (eq 3)	*u*(γ_1_^exp.^)
323.1	12.39	0.01	0.0013	0.0002	5.54	6
323.2	11.69	0.01	0.1053	0.0058	1.80	0.1
333.2	19.95	0.01	0.0012	0.0003	6.63	5
333.3	19.80	0.01	0.0187	0.0028	4.65	0.3
333.2	19.46	0.01	0.0480	0.0050	3.18	0.1
333.2	19.29	0.01	0.0648	0.0056	2.58	0.1
333.3	19.16	0.01	0.0894	0.0068	2.26	0.1
333.2	19.00	0.01	0.1001	0.0067	1.98	0.1
333.2	17.08	0.01	0.2472	0.0116	1.25	0.02
333.2	14.39	0.01	0.3683	0.0180	1.09	0.02
353.2	47.40	0.07	0.0011	0.0006	37.86	5
353.2	46.95	0.07	0.0178	0.0048	19.64	0.4
353.2	46.40	0.07	0.0468	0.0075	11.62	0.2
353.2	46.09	0.07	0.0627	0.0085	9.77	0.1
353.2	45.68	0.07	0.0879	0.0098	7.94	0.1
353.1	45.16	0.07	0.1000	0.0101	7.08	0.1
353.3	41.60	0.07	0.2426	0.0154	4.11	0.06
353.2	35.00	0.06	0.3778	0.0246	3.55	0.05
372.9	99.99	0.16	0.0010	0.0008	16.78	5
373.1	99.91	0.16	0.0169	0.0069	8.31	0.4
373.2	99.79	0.16	0.0435	0.0110	5.17	0.2
373.3	99.56	0.16	0.0606	0.0121	4.07	0.1
373.2	98.68	0.16	0.0847	0.0132	3.13	0.1
373.2	97.75	0.15	0.1001	0.0143	2.85	0.1
373.2	89.93	0.14	0.2483	0.0206	1.52	0.05
373.2	76.59	0.12	0.3982	0.0288	1.13	0.04

a Standard uncertainties u are *u*(*T*) = 0.1K, *u*(*x*_1_) = 0.0002, *u*(*y*_1_) = 0.0002

b The titration method was used to analyze the samples.

**Figure 5:**
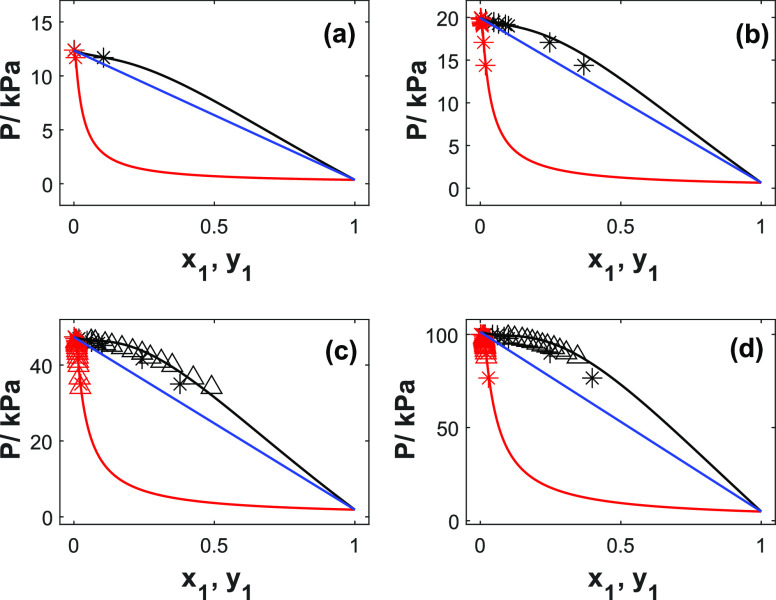
Pressure profiles for the 1-(2HE)PRLD (1)/H_2_O (2) system at different temperatures: (a) 323 K, (b) 333 K, (c) 353 K, and (d) 373 K (∗, this work; Δ, ref (5); solid lines, UNIQUAC; black lines, bubble point lines; red lines; dew point lines; blue lines, Raoult’s law).

The optimum regressed parameters are listed in Table 6, and the parity plots are available in the Supporting Information (Figure S3). Figure 5 gives the UNIQUAC model for 1-(2HE)PRLD (1)/H_2_O (2) together with the binary data from this work and Bernhardsen et al. (2019).^5^ The figure shows that the two data sets agree well, with a slight deviation at high concentrations. The UNIQUAC model predicts well the measured total pressures with an AARD value of 1.7%. The system behaves nonideally, and again, a positive deviation from Raoult’s law is observed for all amine concentrations. The calculated values of the interaction energy (*U*_*ji*_) between the like-molecules and unlike molecules from Table 6 show that the interaction between amine–amine is stronger than that of the amine–H_2_O and H_2_O–H_2_O. The system’s total pressure is mainly due to the water vapor pressure. However, when comparing aqueous solutions of 1-(2HE)PRLD to 2-PPE, it can be seen that the contribution of 1-(2HE)PRLD to the total pressure is higher due to higher volatility. The UNIQUAC model represents well the vapor pressure data with an AARD value of 5.9%. The vapor pressure plots are provided in the Supporting Information (Figure S4). The thermodynamic consistency of the data was checked by first dividing the calculated *y*-values with the experimental *y*-values and, similarly, dividing the calculated *x*-values with the experimental *x*-values.^10,28^ The parity plot in the Supporting Information (Figure S3d) shows good consistency in the data.

**Figure 6:**
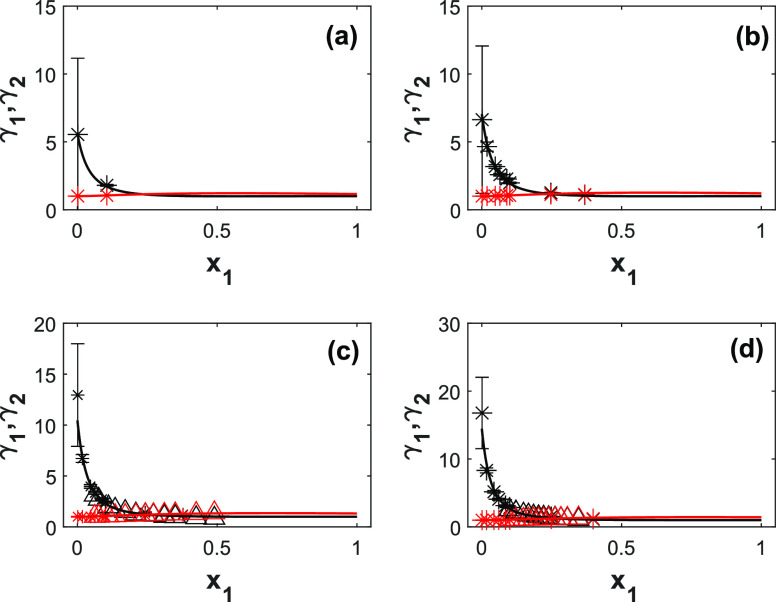
Calculated activity coefficients for 1-(2HE)PRLD (1)/H_2_O (2) at different compositions and temperatures: (a) 323 K; (b) 333 K; (c) 353 K; and (d) 373 K (∗, this work; Δ, ref (5); solid lines, UNIQUAC; black lines, amine; red lines, water).

The UNIQUAC model for amine and water activity coefficients is shown in Figure 6. The model predicts well the activity coefficients with an AARD value of 5.2%. The activity coefficients for both amine and water are weak functions of temperature (see the *b*_*i,j*_ values in Table 6). The amine activity coefficient increases with temperature.

**Figure 7:**
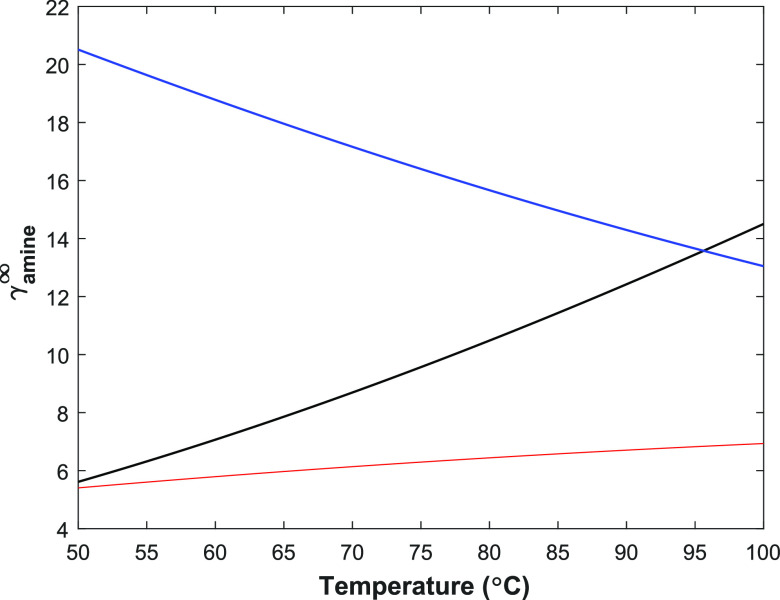
Calculated activity coefficients for 2-PPE and 1-(2HE)PRLD at an infinite dilution as a function of temperature (blue, 2-PPE; black, 1-(2HE)PRLD (this work); red, 1-(2HE)PRLD^5^).

At an infinite dilution, the predicted activity coefficient of 2-PPE decreases from 20 to 12 as temperature increases from 323 to 373 K. An opposite trend is seen for 1-(2HE)PRLD: the activity coefficient increases from 6 to 14 when temperature increases from 323 to 373 K, as shown in Figure 7. The predicted activity coefficient of 1-(2HE)PRLD is lower than that of 2-PPE. At 368 K, the activity of 1-(2HE)PRLD reaches 2-PPE, and at higher temperatures, the activity of 1-(2HE)PRLD is higher than 2-PPE. In Figure 7, activity coefficients at infinite dilution for the 1-(2HE)PRLD measured in this work are compared with the activity coeffients from the literature.^5^ Previously published data clearly show both lower activity coefficients at infinite solutions and more temperature sensitive than the data measured in this work. This is expected since the literature model was developed with limited data for high concentrations and narrow temperature ranges.

## 4 Conclusions

In this work, an ebulliometer measurement of two amine systems is reported. Vapor pressure (boiling point) for pure amines and total pressure over their aqueous solutions up to 0.85 in mass fraction (∼0.44 mole fractions) of 2-PPE and 0.80 in mass fraction (∼0.40 mole fractions) of 1-(2HE)PRLD were measured at different temperatures. Samples were collected at equilibrium, and both liquid and vapor phases were analyzed. The amine concentration was determined by titration or ion chromatography depending on the amine concentration.

The measured substances (2-PPE and 1-(2HE)PRLD) are less volatile than water, but 1-(2HE)PRLD is more volatile than 2-PPE. In the aqueous systems, the amine partial pressure gives a minor contribution to the total pressure of the solutions. The experimental activity coefficients show a weak temperature dependency. In the case of 1-(2HE)PRLD, the activity increases with temperature, whereas the activity of 2-PPE decreases with increasing temperature. Overall, the 1-(2HE)PRLD/H_2_O system is more volatile than the 2-PPE/H_2_O system. Experimental activity coefficients at lower concentrations, particularly at lower temperature, have higher uncertainty compared to the higher concentration data due to low solvent volatility and accuracy of the analytical method used.

The Antoine equation was used to correlate the boiling points of the pure components and together with the UNIQUAC model was used to represent the binary VLE experimental data. The UNIQUAC parameters for size and surface area of amine molecules were estimated with Bondi’s method. The binary interaction parameters were regressed using data for saturation pressure of pure amines, the total pressure of the binary systems, as well as liquid- and vapor-phase compositions. In the regression of the 2-PPE system, excess heat capacity data available in the literature were additionally used. The UNIQUAC model represents well the experimental data.

## Supporting Information Available

Derivation of excess properties: excess enthalpy and heat capacity. Parity plots for the UNIQUAC model for 2-PPE (1)/H_2_O (2): total pressure, saturation pressure, pure amine, vapor-phase composition, excess heat capacity, and ratio of activity coefficient. Graphical presentations of vapor-phase composition for 2-PPE (1)/H_2_O (2) at different liquid compositions and temperatures (333, 343, 353, and 373 K). Parity plots for the UNIQUAC model for 1-(2HE)PRLD (1)/H_2_O (2) total pressure, saturation pressure, activity coefficient, and ratio of activity coefficient. Graphical presentations of the UNIQUAC model for the vapor-phase composition for the 1-(2HE)PRLD (1)/H_2_O (2) system at different temperatures (323, 333, 353, and 373 K) (PDF)
