# Supplementary material for: Vapor–Liquid Equilibria Data for 2-Piperidineethanol and 1-(2-Hydroxyethyl)pyrrolidine in Aqueous Solutions and a UNIQUAC Model Representation
Source: J Chem Eng Data. 2022 Jan 4;67(1):159–66. doi: 10.1021/acs.jced.1c00726 (PMC8762663; doi:10.1021/acs.jced.1c00726)
Supplement: Supplementary file 1 — je1c00726_si_001.pdf [file je1c00726_si_001.pdf]

Vapor liquid equilibria data for 2-Piperidineethanol (2-PPE) and 1-(2-Hydroxyethyl)pyrrolidine (1-(2HE)PRLD) in aqueous solutions and a UNIQUAC model representation

*Ardi Hartono[1], Christina Nøkleby[1], Inna Kim[2], and Hanna K. Knuutila[1]**

1Department of Chemical Engineering, Norwegian University of Science and Technology,

N-7491 Trondheim, Norway

2SINTEF Industry, P.B. 4760, 7465 Trondheim, Norway

* To whom correspondence should be addressed, Phone: +47-73594119, e-mail: hanna.knuutila@ntnu.no

SUPPORTING INFORMATION

- Derivation of excess properties from a calorimetric experiment:
- An excess enthalpy:

$$-\frac{H^E}{R}=\left[\frac{\partial\, G^E/R\cdot T}{\partial T}\right]_{P,x}=\frac{q_1\cdot x_1\cdot\theta_2\cdot\tau_{21}\cdot b_{21}}{(\theta_1+\theta_2\cdot\tau_{21})}+\frac{q_2\cdot x_2\cdot\theta_1\cdot\tau_{12}\cdot b_{12}}{(\theta_2+\theta_1\cdot\tau_{12})}$$

- An excess heat capacity:

$$C_p^E=\frac{\partial H^E}{\partial T}=R\cdot\theta_1\cdot\theta_2\cdot\left\{q_1\cdot x_1\cdot\frac{b_{21}\cdot\tau_{21}\cdot b_{21}}{(\theta_1+\theta_2\cdot\tau_{21})^2}+q_2\cdot x_2\cdot\frac{b_{12}\cdot\tau_{12}\cdot b_{12}}{(\theta_2+\theta_1\cdot\tau_{12})^2}\right\}$$

LIST OF FIGURES

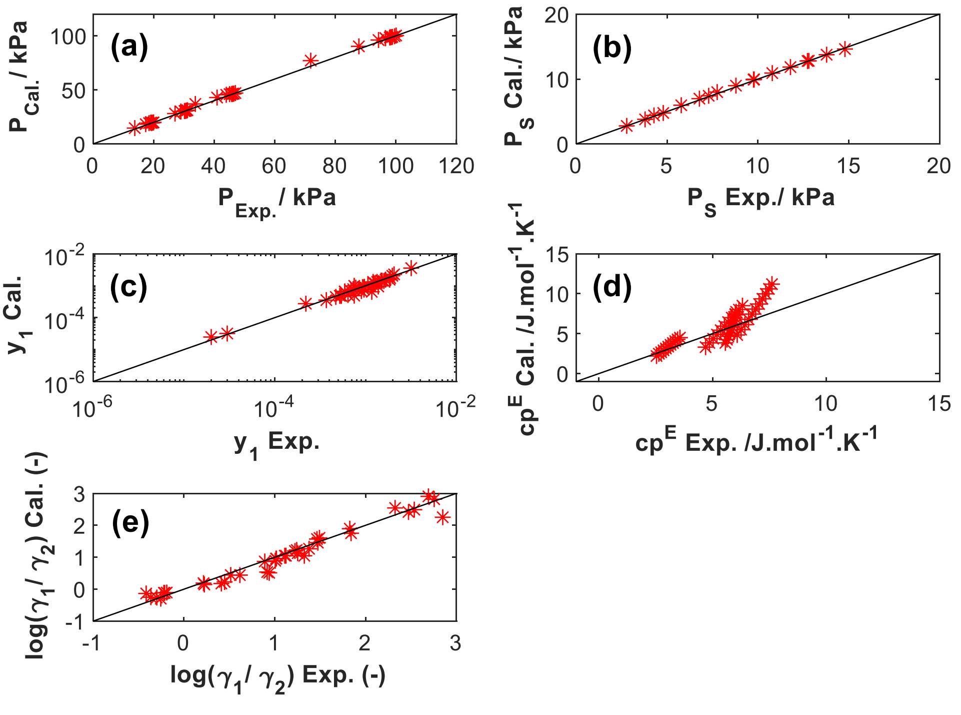

Figure S1. Parity plots for the UNIQUAC model for 2-PPE(1)/H2O(2) ((a). Total pressure (P_i) (b). Saturation pressure pure amine (P_i^S), (c). Vapor phase composition (Y_i), (d). Excess heat capacity (Cp_i^E), (e). Ratio of activity coefficient.

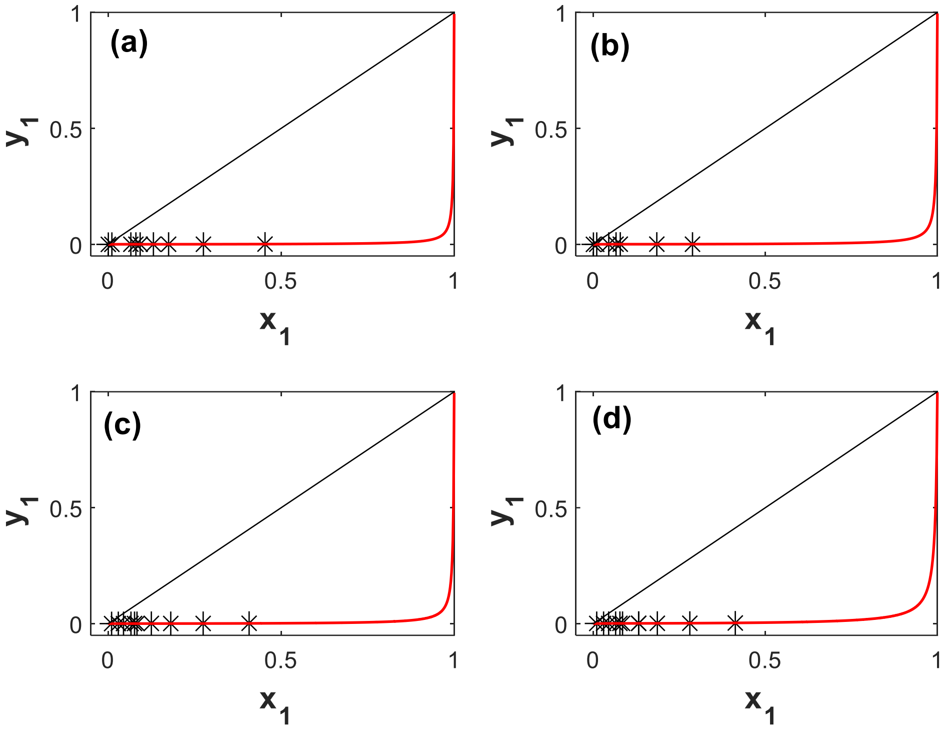

Figure S2. Vapor phase composition for 2-PPE(1)/H2O(2) at different liquid compositions and temperatures ((a). 333K (b). 343K (c). 353K (d). 373K (Points, data; Solid lines, UNIQUAC).

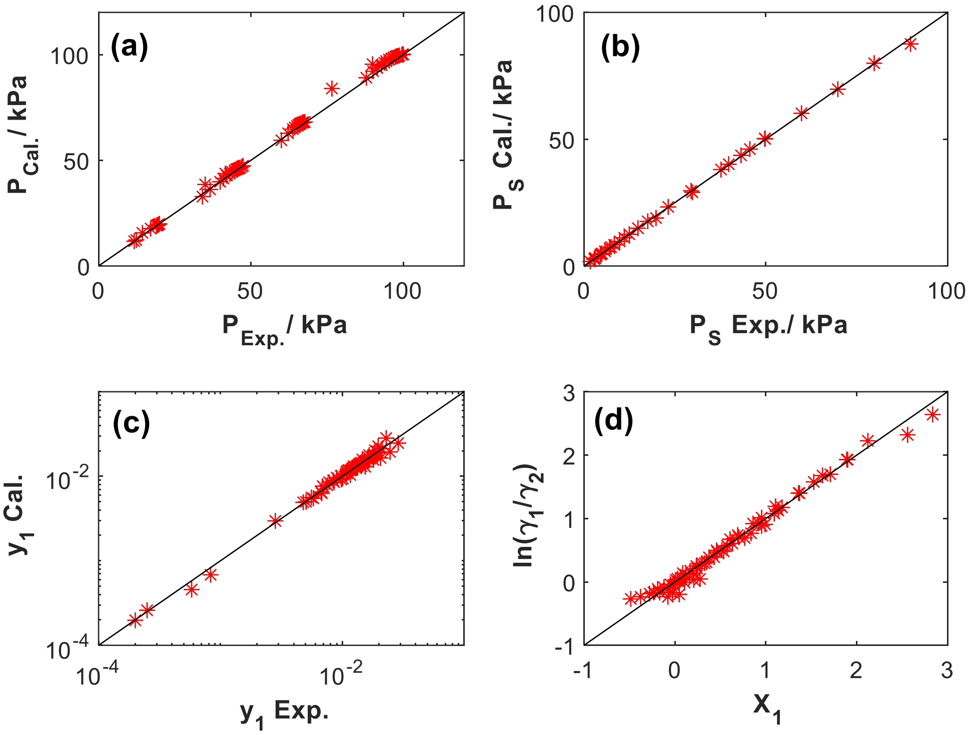

Figure S3. Parity plots for the UNIQUAC model of 1-(2HE)PRLD(1)/H2O(2) ((a). Total pressure (P_i) (b). Saturation pressure pure amine $\left(P_i^S\right)$, (c). Activity coefficient (γ_i), (d). Ratio of activity coefficient.

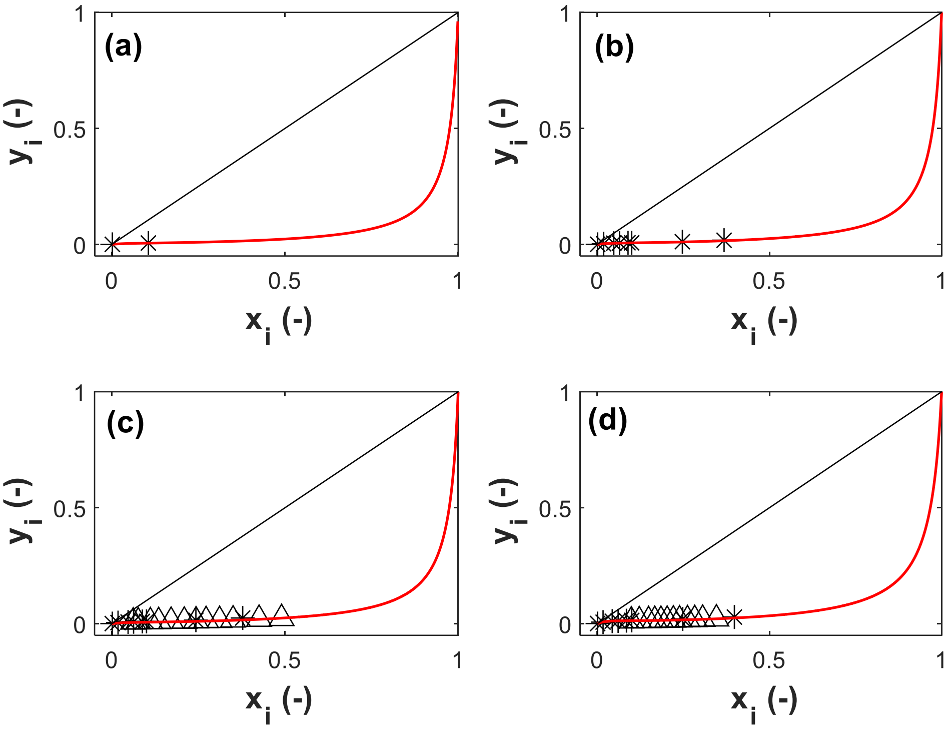

Figure S4. The UNIQUAC model for the vapor phase composition for the 1-(2HE)PRLD(1)/H2O(2) system at different temperatures: (a). 323K (b). 333K (c). 353K (d). 373K (✳, This work; △, (5); Solid lines, UNIQUAC; Black lines, Amine; Red lines; Water).
